# Case Report: Effective methotrexate removal by combined hemodialysis and polymeric resin hemoadsorption

**DOI:** 10.3389/fneph.2025.1644079

**Published:** 2025-08-25

**Authors:** Maria Rita Dias, Carla Nicolau, Hugo Ferreira, Sérgio Chacim, Isabel Oliveira, Gonçalo de Câmara Negalha, José Mário Mariz, José Maximino Costa

**Affiliations:** ^1^ Department of Nephrology, Hospital Garcia de Orta, Unidade Local de Saúde de Almada-Seixal, Almada, Portugal; ^2^ Department of Nephrology, Hospital Curry Cabral, Unidade Local de Saúde de São José, Lisboa, Portugal; ^3^ Department of Nephrology, Instituto Português de Oncologia do Porto Francisco Gentil, Porto, Portugal; ^4^ Department of Hematology, Instituto Português de Oncologia do Porto Francisco Gentil, Porto, Portugal

**Keywords:** methotrexate, high-dose methotrexate, drug toxicity, acute kidney injury, hemodialysis, hemoadsorption, hemoperfusion, HA230

## Abstract

**Background:**

High-dose methotrexate (HDMTX) is central to treating primary central nervous system lymphoma but carries a risk of acute kidney injury (AKI), which can delay methotrexate (MTX) clearance and increase toxicity. Glucarpidase is the treatment of choice for MTX toxicity, but limited access in many countries may necessitate alternatives. We present the first reported adult case of combined high-flux hemodialysis (HFHD) and HA230 hemoadsorption for MTX clearance.

**Case summary:**

A 66-year-old woman with newly diagnosed primary central nervous system lymphoma began induction chemotherapy including HDMTX. Forty-eight hours post-infusion, she developed KDIGO stage 3 AKI, with plasma MTX levels of 26.278 µmol/L despite maintained urine output and early supportive measures. On Day 3, MTX levels remained elevated at 15.567 µmol/L, accompanied by severe metabolic alkalosis. She was admitted to intensive care, where she underwent HFHD combined with post-filter HA230 hemoadsorption, followed by intravenous glucarpidase as soon as it became available. A second extracorporeal session occurred 48 hours later. MTX levels decreased by 91.93% (estimated elimination half-life ≈ 0.83 hours) and 71.02% (half-life ≈ 2.12 hours) after the first and second sessions, respectively. No significant rebound in MTX levels or dialysis-related complications occurred. The patient recovered renal function and completed further treatment without MTX.

**Conclusions:**

This case demonstrates the effectiveness of combined HFHD and HA230 hemoadsorption as a bridging or alternative strategy when glucarpidase is delayed or unavailable. While evidence remains limited, it supports further investigation into extracorporeal MTX removal and contributes to the evolving field of Onconephrology.

## 1 Introduction

Methotrexate (MTX) is an antimetabolite chemotherapy agent widely used at high doses (≥500 mg/m² intravenously) as a key component of current remission induction protocols for primary central nervous system lymphoma (1). Despite its efficacy, high-dose MTX (HDMTX) is associated with hepatic, renal, mucosal, neurological, and hematological toxicity (2).

Approximately 90% of MTX is excreted unchanged in the urine (3). At high concentrations in acidic urine, it can precipitate as crystals within the renal tubules, leading to tubular injury (2, 3). HDMTX-associated acute kidney injury (AKI) delays MTX clearance, thereby increasing the risk of systemic toxicity and mortality (3, 4). AKI also prevents administration of further MTX doses, thereby limiting effective treatment of the underlying malignancy (2, 4).

Despite supportive care measures during administration of HDMTX, AKI occurs in approximately 2%–12% of patients (5). Risk factors include dehydration, acidic urinary pH, persistent elevations in plasma MTX concentration, hypoalbuminemia, third-space fluid collections, concurrent use of nephrotoxic drugs, preexisting renal or hepatic dysfunction, elevated body mass index (BMI), and polymorphisms in genes involved in MTX absorption, metabolism, excretion, cellular transport, or target pathways (notably *MTHFR* and *ABCB1*) (2, 6–8).

HDMTX-associated AKI is typically non-oliguric and often reversible (3), although 1%–10% of patients may require renal replacement therapy (4). Serum creatinine typically peaks within the first week and returns to baseline within 1–3 weeks (4, 9). However, up to 10% of patients may exhibit reduced glomerular filtration rate at three months (4).

Preventive strategies include MTX dose adjustment to kidney function, aggressive hydration, urine alkalinization, and rescue therapy with leucovorin to neutralize the effects of MTX (2, 10). Monitoring of serum creatinine, electrolytes, and MTX concentrations is essential during HDMTX therapy (2, 10).

The preferred treatment is glucarpidase, a recombinant carboxypeptidase that rapidly cleaves MTX into inactive metabolites, 2,4-diamino-N10-methylpteroic acid (DAMPA) and glutamate (10). However, access to glucarpidase is limited in many countries due to its high cost (10).

In such cases, extracorporeal techniques may serve as a bridge or as an alternative (4, 11, 12). High-flux hemodialysis (HFHD) is the most effective dialysis modality for MTX removal, although its efficacy may be limited for protein-bound or intracellular MTX fractions (3, 4). While MTX has a low molecular weight (454 Da), it is approximately 50% protein-bound and has a large extravascular volume of distribution, making it only moderately dialyzable (4).

The HA230 cartridge (Jafron^®^) is a polymeric resin hemoadsorber with affinity for protein-bound solutes, targeting molecules within a pore size range of 200 Da to 10 kDa. It has demonstrated efficacy in removing highly protein-bound drugs (13, 14). Its primary indications include poisoning from herbicides, rodenticides, pesticides, biotoxins, and drug overdoses. It may also be considered for the removal of excessive cytostatic agents from the bloodstream (13). The cartridge can be used alone or in conjunction with intermittent or continuous hemodialysis (HD), either in pre- or post-filter configurations (15, 16).

This report describes the first adult case of combined HFHD and HA230 hemoadsorption for MTX clearance following AKI, demonstrating rapid drug elimination and favorable clinical outcomes.

## 2 Case report

A 66-year-old Caucasian female (weight: 63 kg; BMI: 26.2 kg/m²; body surface area: 1.65 m^2^) presented with confusion without motor or sensory deficits. Her medical history included breast carcinoma (treated 7 years earlier with neoadjuvant chemotherapy, surgery, adjuvant radiotherapy, and hormonal therapy), a 6-year history of hypertension, past smoking (35 pack-years), and childhood appendicectomy. She had no known drug allergies.

Magnetic Resonance Imaging (MRI) and Positron Emission Tomography (PET) scans revealed two cerebral lesions (right posterior parasagittal and left frontal), without extracranial involvement. Methylprednisolone 32 mg was initiated with symptom improvement. An excisional biopsy confirmed the diagnosis of diffuse large B-cell lymphoma. Three months later, she was admitted for chemotherapy. Her home medications on admission included levetiracetam 500 mg, methylprednisolone 4 mg (weaned over the previous 3 months), bisoprolol 1.25 mg, and omeprazole 20 mg. Repeat MRI showed two different lesions (splenium of corpus callosum and right frontal parasagittal), suggesting disease progression. Bone marrow biopsy was negative. Her baseline serum creatinine was 78 µmol/L and albumin was 33 g/L.

The patient was initiated on prophylactic urine alkalinization with intravenous (IV) sodium bicarbonate 1.4% q4h prior to starting R-MPV chemotherapy (rituximab, MTX, procarbazine, and vincristine). On the first day, IV rituximab 825 mg (500 mg/m²) and methylprednisolone 80 mg were administered. The following day, the patient underwent a lumbar puncture (negative for neoplastic cells), followed by administration of intrathecal MTX 12 mg, IV MTX 5775 mg (3500 mg/m²) over 2 hours, IV vincristine 2 mg (≈1.4 mg/m²), and oral procarbazine 100 mg/m²/day (planned for 7 days). This day is designated Day 0.

During the first 24 hours post-MTX, her urine output was approximately 1 L. IV furosemide 20 mg was given with good diuretic response. Urine pH was monitored every 6 hours and fell below 7 once, requiring correction with IV sodium bicarbonate 8.4%. IV leucovorin 15 mg/m² q6h was started at 24 hours post-MTX.

At 48 hours post-MTX, serum MTX – measured using the Architect Methotrexate chemiluminescence assay (Abbott Diagnostics, IL, USA) on the Architect 2P49 system (Abbott Diagnostics) – was 26.278 µmol/L and creatinine had risen to 273 µmol/L. Urine output was >3 L/day. Severe metabolic alkalosis and hypokalemia were noted (arterial blood gas analysis: pH 7.62, pCO_2_ 61 mmHg, bicarbonate 62.7 mmol/L, potassium 3.2 mmol/L, lactate 1.2 mmol/L). The patient had generalized edema (11 kg weight gain) and hypoxemia (oxygen saturation 80% on room air), along with tremors (without fever) and elevated inflammatory markers. Management included increased leucovorin dosing (100 mg/m^2^ IV q6h), IV potassium chloride bolus (40 mEq), furosemide 20 mg IV q6h, oxygen via nasal cannula (4 L/min), and empirical piperacillin/tazobactam.

By Day 3, MTX levels remained elevated at 15.567 µmol/L, creatinine reached 300 µmol/L and alkalosis worsened (pH 7.66, bicarbonate 67.6 mmol/L), prompting referral to Nephrology. Urinalysis showed 5–10 erythrocytes per high-power field with no other abnormalities. Chest computed tomography showed bilateral pleural effusions and underlying emphysema.

**Figure 1:**
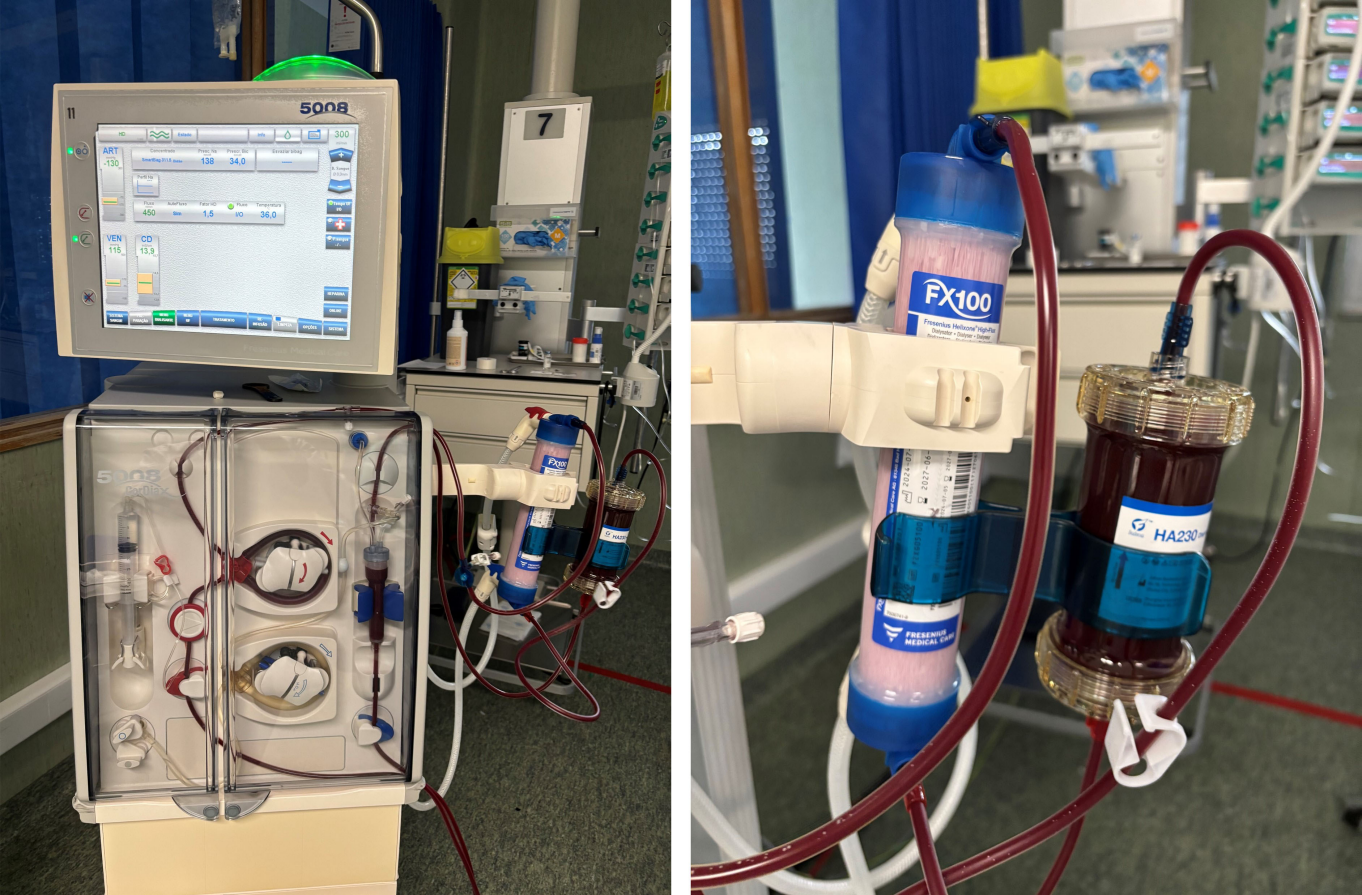
Photographs of the extracorporeal circuit setup using the Fresenius 5008^®^ system and HA230 hemoadsorption cartridge. The HA230 cartridge is placed post-filter in the circuit.

She was transferred to the Intensive Care Unit (ICU) and started on HFHD via a right femoral catheter. The procedure was performed using a Fresenius 5008^®^ machine with an FX100 dialyzer and a HA230 hemoadsorption cartridge positioned post-filter (**Figure 1**), following heparinization with 12,500 units of unfractionated heparin (UFH) injected into the cartridge (reduced dose due to thrombocytopenia). Smartbag 311.5 was used as dialysate (temperature 36°C, bicarbonate 32 mmol/L, target sodium 138 mmol/L). Blood flow rate was 300 mL/min, dialysate flow rate was set at 450 mL/min (AutoFlow factor of 1.5), and total ultrafiltration volume was 1000 mL. Circuit anticoagulation consisted of 2,000 units of UFH administered at initiation, with an additional 2,000 units given during the session; however, the circuit clotted after 3 hours. IV glucarpidase 3,000 units (≈50 units/kg) was given post-dialysis as soon as it became available. Leucovorin was held 2 hours before and resumed 2 hours after glucarpidase.

**Figure 2:**
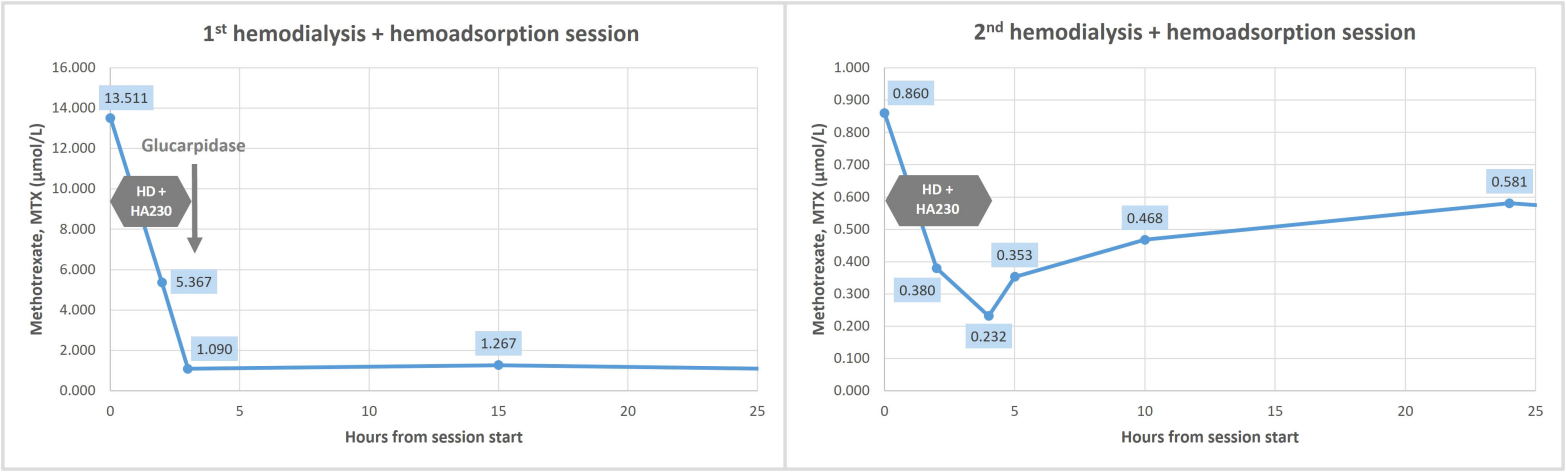
Serum methotrexate (MTX) level kinetics during and after the first and second combined sessions of high-flux hemodialysis and hemoadsorption using HA230 cartridge. HD, hemodialysis.

**Figure 3:**
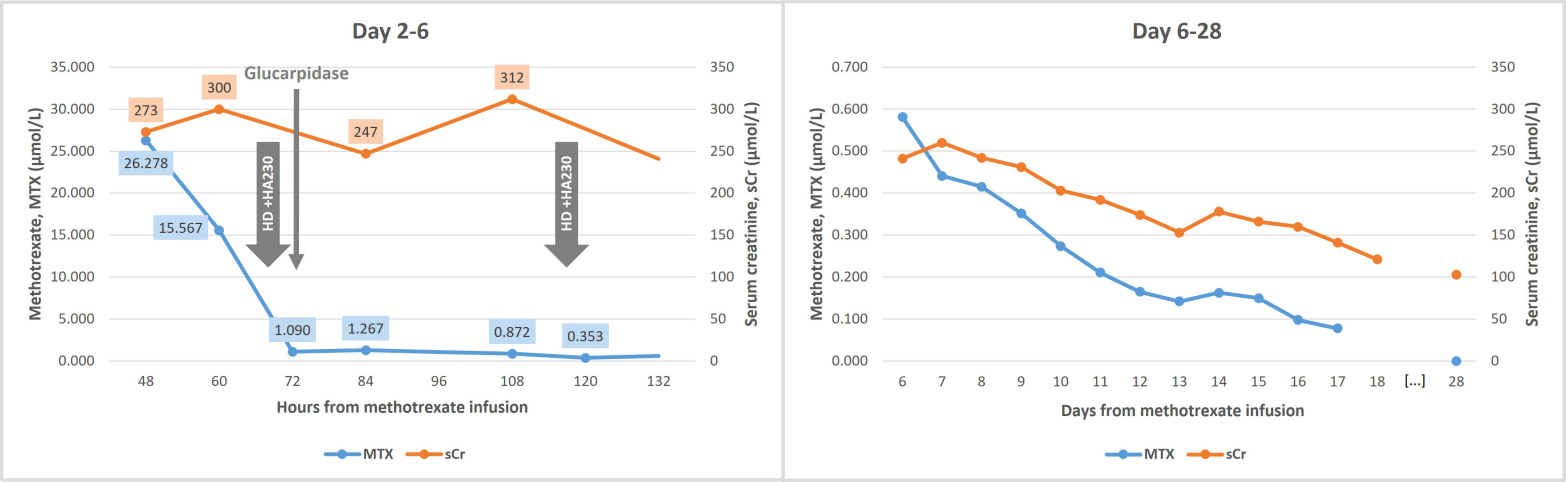
Serum methotrexate (MTX) and creatinine (sCr) level kinetics throughout hospitalization and at the first follow-up visit (Day 28). HD, hemodialysis.

The patient remained in the ICU for four days, during which she maintained hemodynamic stability and preserved urine output with hypervolemia and metabolic alkalosis correction (**Table 1**). Oxygen was weaned to room air. A second HFHD/hemoadsorption session was completed on Day 5, 48 hours after the first session and glucarpidase administration, at which point MTX level remained at 0.872 µmol/L. The 4-hour session was performed without ultrafiltration, following heparinization with the recommended 25,000 units of UFH administered into the cartridge (17). Circuit anticoagulation was adjusted to 2,500 units of UFH at initiation and an additional 2,500 units during the session. All other settings were similar to the previous session. **Figures 2** and **3** show changes in MTX levels and serum creatinine during and after each session. The patient continued sodium leucovorin. Other MTX toxicities were limited to transient mild mucositis, as well as reversible hepatotoxicity, anemia, and thrombocytopenia (**Table 1**). Cultures remained negative after 7 days of antibiotic therapy.

**Table 1:** Serum studies in the first week of hospitalization.

Laboratory blood parameters		Reference levels	Day 0	Day 2	Day 3	Day 4	Day 5	Day 6	Day 7
MTX (µmol/L)		<0.02	-	26.278	13.511	1.267	0.872	0.581	0.441
Creatinine (µmol/L)		45-84	55	273	300	247	312	241	260
eGFR (mL/min/1.73 m²)		>90	>90	15	13.44	16.97	13	18	16
Urea (mmol/L)		1.6-8.3	2.8	10.4	10	6.9	10.3	8.3	10
Sodium (mmol/L)		135-145	143	143	144	139	140	140	142
Potassium (mmol/L)		3.8-5	3	3.1	3.2	4.1	3.7	3.4	3.2
Calcium (mmol/L)		2.2-2.65	2.16	2	2.02	2.19	2.22	2.3	2.21
ABG	pH	7.35-7.45	–	7.62	7.66	7.51	7.48	7.47	7.44
	HCO3^-^ (mmol/L)	22-26	-	62.7	67.6	36.7	35	32	31.2
	pCO2 (mmHg)	35-45	–	61	60	46	47	44	46
	pO2 (mmHg)	80-100	-	66	66	81	74	96	-
	Lactate (mmol/L)	0.5-1.6	–	1.2	1.2	1.1	0.6	0.5	0.8
Hemoglobin (g/L)		115-165	119	93	112	95	104	101	99
Platelets (x10^9^/L)		150-450	118	62	74	64	69	56	47
Leukocytes (x10^9^/L)		4-11	11.14	5.76	6.77	5.58	5.27	2.87	5.7
C-reactive protein (mg/L)		<5	9.7	161.9	144.9	98.5	54.8	38.6	22.4
Albumin (g/L)		38-53	33	31	29	26	28	28	30
Total bilirubin (µmol/L)		<17	12.3	30.2	36.7	26.1	26.2	21.4	16.8
Direct bilirubin (µmol/L)		<4.3	2.2	-	9.4	9.9	10	6.9	4.7
AST (U/L)		<39	22	94	77	49	37	32	30
ALT (U/L)		<42	23	143	114	83	68	56	44
ALP (U/L)		42-128	40	–	54	57	66	79	80
GGT (U/L)		7-52	18	70	70	84	110	126	131
LDH (U/L)		67-248	257	459	445	333	319	274	297

ABG, arterial blood gas analysis; ALP, alkaline phosphatase; ALT, alanine transaminase; AST, aspartate transaminase; eGFR, estimated glomerular filtration rate (according to 2009 CKD-EPI equation); GGT, gamma-glutamyl transferase; HCO3^-^, bicarbonate; LDH, lactate dehydrogenase; MTX, methotrexate; pCO2, partial pressure of carbon dioxide; pO2, partial pressure of oxygen. Combined hemodialysis and hemoadsorption sessions were performed on Days 3 and 5, following the laboratory results.

Following ICU discharge, the patient returned to the Hematology ward. MTX levels declined and kidney function improved (**Figure 3**) with IV hydration and continued leucovorin. Urine pH was checked daily and remained >7. She had an episode of febrile neutropenia treated with a repeat 7-day course of piperacillin/tazobactam. She was discharged on Day 18 with a serum creatinine of 121 µmol/L, estimated glomerular filtration rate (eGFR) of 40.3 mL/min/1.73 m² (according to 2009 CKD-EPI equation), and MTX level of 0.078 µmol/L.

At follow-up one month later, she had a stable eGFR of 45 mL/min/1.73 m² (serum creatinine 103 µmol/L) and minimal proteinuria (urinary protein/creatinine and albumin/creatinine ratios of 0.1 g/g and 31.4 mg/g, respectively). Given the previous toxicity, the Hematology team elected to continue the R-MPV protocol, omitting MTX and including procarbazine in every cycle. At 2 months post-MTX, her eGFR had improved to 59 mL/min/1.73 m² (serum creatinine 88 µmol/L). Following the fifth cycle of chemotherapy (without MTX), PET was negative and brain MRI demonstrated a partial response. She therefore underwent a total of seven chemotherapy cycles, followed by whole-brain radiotherapy, which was completed five months post-MTX. At this time, her eGFR remained stable at 72 mL/min/1.73 m² (serum creatinine 75 µmol/L), with no abnormalities on urinalysis and no proteinuria. At the time of writing, she was awaiting consolidation chemotherapy with cytarabine.

## 3 Discussion

This case illustrates the successful use of combined HFHD and HA230 hemoadsorption in managing severe MTX toxicity in an adult. To our knowledge, this is the first adult case and the second overall to report the use of the HA230 cartridge for this indication.

Plasma MTX concentrations above 10 µmol/L at 24 hours, 1 µmol/L at 48 hours, or 0.1 µmol/L at 72 hours are associated with increased systemic toxicity risk (3). Our patient exceeded these thresholds and developed KDIGO stage 3 AKI (18). Contributing factors included a high BMI, hypoalbuminemia, transient urine pH below 7, and suboptimal diuresis on Day 1 – less than the desired ≥2.5 L/m^2^/day (3, 10), a target intended to reduce MTX concentration in the tubular fluid. Additionally, pleural effusions likely perpetuated elevated MTX levels. We also acknowledge that obtaining the first serum MTX measurement at 48 hours rather than the recommended 24-hour mark may have delayed diagnosis and prolonged exposure to toxic MTX levels. This deviation from the local protocol – which advises daily MTX monitoring – was likely an isolated oversight.

A variety of MTX removal strategies have been employed – including peritoneal dialysis, HD, HFHD, hemodiafiltration, continuous venovenous HD and hemodiafiltration (CVVHDF), charcoal hemoadsorption, and plasma exchange – although their overall effectiveness remains limited (4, 12). Among these, HD demonstrates the greatest median reduction in plasma MTX concentrations (76%) and shortest elimination half-life (*t*_½_ ≈ 4 hours) per session. However, it cannot effectively remove protein-bound or intracellular MTX, often resulting in significant post-dialysis rebound (3, 4, 19). Because MTX reduction depends on session duration, *t½* is a more reliable parameter than percentage clearance (4, 11). It can be calculated using the formula *t*_½_ = (ln 2×t)/ln(C_0_/C_t_), where *t* is the session duration, *C_0_* the initial MTX concentration and *C_t_* the final concentration (11, 20).

The HA230 cartridge (13, 14), a resin-based hemoadsorber capable of binding solutes up to 10 kDa – including protein-bound drugs – was previously used in a pediatric leukemia case (16). In that report, a single 4-hour session combined with CVVHDF achieved an 85.27% MTX reduction (*t_½_* = 1.45 hours) (16). In our case, MTX levels fell by 91.93% after the first session and by 71.02% after the second, with calculated *t_½_* of 0.83 and 2.12 hours, respectively – shorter than those reported with other modalities. No significant post-treatment rebound or procedural complications occurred.

Nevertheless, glucarpidase remains the standard of care, rapidly hydrolyzing over 95% of both free and protein-bound intravascular MTX within 15 minutes (*t_½_* ≈ 3 minutes) (4, 10). However, its limited availability and high cost restrict global access. In Portugal, prior authorization is required, potentially delaying administration, as occurred in our case. Additionally, it introduces DAMPA, an inactive metabolite that interferes with immunoassay-based MTX measurements for at least 48 hours, leading to falsely elevated readings – during this period, MTX concentrations can only be accurately measured using chromatographic methods (10, 21). These limitations, combined with the potential for MTX rebound and the inability to repeat dosing (4, 10), underscore the need for alternative or complementary strategies. In our case, due to severe refractory metabolic alkalosis and anticipated delay in glucarpidase delivery, HFHD was initiated promptly. Post-glucarpidase, extracorporeal therapy was resumed at 48 hours due to persistently elevated MTX levels, acknowledging potential immunoassay interference from the DAMPA metabolite. We were unable to quantify DAMPA levels or confirm MTX concentrations using chromatographic methods due to limited availability of this technique in Portugal.

Despite their utility, high-quality evidence supporting both glucarpidase and extracorporeal techniques remains limited and robust data on clinical benefit, such as mortality reduction, are lacking. Much of the existing literature is derived from case reports or small series (4, 10, 12, 16). In a 2022 systematic review, the EXTRIP Workgroup itself evaluated the available evidence and issued a strong recommendation against the use of extracorporeal treatments as alternatives or adjuncts to glucarpidase, citing limited supporting data (4). However, the workgroup acknowledged a potential benefit in rare cases with a large intravascular MTX burden, although glucarpidase would be preferred if available. A target concentration below 0.1 µmol/L was considered a reasonable threshold for discontinuing extracorporeal therapy (4) – in our case, it was stopped earlier based on clinical improvement and resolving AKI.

Supportive measures should be continued despite glucarpidase or extracorporeal MTX removal, as intracellular MTX is not eliminated by either method (2, 4). Importantly, leucovorin is both dialyzable and metabolized by glucarpidase, so it should be administered strategically around extracorporeal sessions and glucarpidase administration to maintain therapeutic effect (2, 4), as was done in our patient.

Rechallenge with MTX following toxicity remains controversial. Data from two small cohorts – one involving 20 children (22) and the other 11 adults with lymphoma (23) – suggest that, with dose reductions and close monitoring, rechallenge may be feasible. In our case, however, MTX was omitted from subsequent treatment cycles to prioritize patient safety; fortunately, the patient still achieved a partial response. Additionally, although pharmacogenetic testing (e.g., for MTHFR 677C>T polymorphism) is not currently standard practice and was not performed in this case, emerging data suggest it may help individualize HDMTX dosing and reduce toxicity (8).

Despite the favorable clinical course observed in our patient up to five months post-MTX, longer-term outcomes remain unknown. We continue to monitor the patient’s clinical status and renal function, and consider future follow-up reports as more data become available.

This report is inherently limited by its single-case design, precluding statistical analysis or measures of variability. The MTX elimination half-lives we calculated offer a comparative reference but are based on a simplified model assuming linear pharmacokinetics. However, in the absence of pharmacokinetic studies on combined hemodialysis and HA230 hemoadsorption, nonlinear elimination cannot be excluded. Future prospective case series, registries, or multi-center studies are needed to better define treatment efficacy, pharmacokinetics, and inter-patient variability. Moreover, chromatographic methods should be prioritized for MTX monitoring after glucarpidase, particularly in research settings.

Additionally, the cost-effectiveness and accessibility of HA230 hemoadsorption warrant thoughtful evaluation. Although glucarpidase is highly effective, its substantial cost and limited availability constrain its widespread use. In contrast, HA230 cartridges are generally more accessible and considerably less expensive. Nevertheless, overall costs may increase when multiple hemoadsorption sessions are required, particularly in combination with hemodialysis. Future pharmacoeconomic studies are needed to assess the feasibility and value of this approach across various healthcare settings, considering factors such as hospital length of stay, staffing requirements, and equipment utilization.

In conclusion, HDMTX-associated toxicities, particularly KDIGO stage 3 AKI, can significantly impact patient morbidity, mortality, and subsequent cancer treatment decisions. In cases of severe MTX toxicity complicated by AKI – especially when glucarpidase is unavailable or delayed – combined HFHD and HA230 hemoadsorption may represent a viable strategy to enhance MTX clearance and mitigate systemic toxicity. However, larger, controlled studies are needed to better define the role of HA230 in this context and to guide its integration into the evolving field of Onconephrology.

## Data Availability

The original contributions presented in the study are included in the article/supplementary material. Further inquiries can be directed to the corresponding author.
